# Neighbor-Dependent Ramachandran Probability Distributions of Amino Acids Developed from a Hierarchical Dirichlet Process Model

**DOI:** 10.1371/journal.pcbi.1000763

**Published:** 2010-04-29

**Authors:** Daniel Ting, Guoli Wang, Maxim Shapovalov, Rajib Mitra, Michael I. Jordan, Roland L. Dunbrack

**Affiliations:** 1 Department of Statistics, University of California Berkeley, Berkeley, California, United States of America; 2 Institute for Cancer Research, Fox Chase Cancer Center, Philadelphia, Pennsylvania, United States of America; Duke University, United States of America

## Abstract

Distributions of the backbone dihedral angles of proteins have been studied for over 40 years. While many statistical analyses have been presented, only a handful of probability densities are publicly available for use in structure validation and structure prediction methods. The available distributions differ in a number of important ways, which determine their usefulness for various purposes. These include: 1) input data size and criteria for structure inclusion (resolution, R-factor, etc.); 2) filtering of suspect conformations and outliers using B-factors or other features; 3) secondary structure of input data (e.g., whether helix and sheet are included; whether beta turns are included); 4) the method used for determining probability densities ranging from simple histograms to modern nonparametric density estimation; and 5) whether they include nearest neighbor effects on the distribution of conformations in different regions of the Ramachandran map. In this work, Ramachandran probability distributions are presented for residues in protein loops from a high-resolution data set with filtering based on calculated electron densities. Distributions for all 20 amino acids (with cis and trans proline treated separately) have been determined, as well as 420 left-neighbor and 420 right-neighbor dependent distributions. The neighbor-independent and neighbor-dependent probability densities have been accurately estimated using Bayesian nonparametric statistical analysis based on the Dirichlet process. In particular, we used hierarchical Dirichlet process priors, which allow sharing of information between densities for a particular residue type and different neighbor residue types. The resulting distributions are tested in a loop modeling benchmark with the program Rosetta, and are shown to improve protein loop conformation prediction significantly. The distributions are available at http://dunbrack.fccc.edu/hdp.

## Author Summary

The three-dimensional structure of a protein enables it to perform its specific function, which may be catalysis, DNA binding, cell signaling, maintaining cell shape and structure, or one of many other functions. Predicting the structures of proteins is an important goal of computational biology. One way of doing this is to figure out the rules that determine protein structure from protein sequences by determining how local protein sequence is associated with local protein structure. That is, many (but not all) of the interactions that determine protein structure occur between amino acids that are a short distance away from each other in the sequence. This is particularly true in the irregular parts of protein structure, often called loops. In this work, we have performed a statistical analysis of the structure of the protein backbone in loops as a function of the protein sequence. We have determined how an amino acid bends the local backbone due to its amino acid type and the amino acid types of its neighbors. We used a recently developed statistical method that is particularly suited to this problem. The analysis shows that backbone conformation prediction can be improved using the information in the statistical distributions we have developed.

## Introduction

The empirical distributions of the backbone dihedral angles φ and ψ of amino acids in proteins have been studied for over 40 years. Early efforts were based on determining those regions of the Ramachandran map that are “allowed” and those that are forbidden due to steric conflicts among the backbone atoms or between backbone and the Cβ carbon atom of side chains [1]. This steric analysis has recently been updated and refined by Ho et al. [2], [3]. Boundaries between populated and unpopulated regions have been used as checks on the quality of newly determined experimental structures in such programs as Procheck [4], Whatcheck [5], and more recently MolProbity [6]. Because bond lengths and bond angles vary to only a limited extent (although more so than is typically assumed [7]), protein structures are often treated with only dihedral degrees of freedom in simulations, structure prediction, and structural analysis. Ramachandran data therefore play a central role in developing empirical energy functions for structure prediction [8] and simulation [9].

We distinguish two concepts in analyzing the backbone dihedral angles of proteins. The first is a *Ramachandran plot* or *Ramachandran map*, which is simply a scatter plot of the φ,ψ values for the amino acids in a single protein structure or a set of protein structures. It may be restricted to a single amino acid type and/or a single structural feature type, such as protein loops. The second is a *Ramachandran distribution*, which we use here to mean a statistical representation of Ramachandran data, usually in the form of a probability density function (N.B. by *distribution*, we do *not* mean the cumulative distribution function or CDF). A probability density function gives the probability of finding an amino acid conformation in a specific range of φ,ψ values. For instance, if the function is given on a 10°×10° grid from −180° to +180° in φ,ψ (1296 values), then the distribution may give the probability per 10°×10° region. It could also be expressed per degree squared or per radian squared. Such distributions may be derived for specific amino acid types and/or for specific structural features.

There are several important considerations in developing Ramachandran distributions from structural data, depending on the purpose of the derived distribution. First, while glycine and proline are usually treated separately, the other 18 amino acids are often treated as a single type. However, these amino acids are quite different in their proportions of residues in the α, β, polyproline II, and left-handed helical regions. Second, quite different distributions are determined when either all residues are used or only those outside the regular secondary structures of α-helices and β-sheets [10]. The latter are often assumed to be “intrinsic” preferences of the backbone [11], not influenced by forming specific hydrogen bonds present in regular secondary structures. Third, the quality and quantity of the data are crucial in determining distributions meant to act as quality filters for newly determined structures or for structure prediction. As more structures have become available at higher resolutions, it is now possible to use quite large datasets with resolution cutoffs of 1.8 Å or even better. Other filters have been used including B-factors and steric clashes to remove residues that may be modeled improperly or at least with considerable uncertainty within the electron density. For instance, by using higher resolution structures, B-factors, steric overlaps, and other checks, Lovell et al. [12] were able to determine Ramachandran distributions with smaller “allowed” and “generously allowed” regions than previous efforts. Fourth, most previous efforts have involved density estimation using simple histogram methods – the counts or proportion of counts of residues in non-overlapping square bins of the φ,ψ space. However, even when a large number of proteins are used, the distribution in φ,ψ space may be quite bumpy.

It is therefore of some importance to use a proper density estimation method that results in smooth distributions and minimizes the effects of outliers. This has been accomplished in a number of ways [12], [13], [14], [15]. The Richardson group used kernel density estimates to obtain Ramachandran distributions for Gly, pre-Pro, and non-Gly,non-pre-Pro residues [12]. Kernel density estimates are performed by placing a kernel function such as a Gaussian on each data point, and the density estimate is produced on a grid by summing up the values of all of the kernel functions across the data. Although not described as such, they used what in effect are *adaptive* kernel density estimates [16], [17], such that the data are smoothed to a greater extent with wider kernel functions in sparsely populated regions of the space, while in more populated regions, narrower kernel functions can be used. Because they used a narrow kernel and grouped all non-Gly, non-Pro, non-pre-Pro residues together, the resulting distributions are well-suited to structure validation. Amir et al. used non-adaptive kernel density estimates, but with removal of outliers and addition of pseudocounts in sparsely populated regions [13]. To provide smoothness and differentiability, they calculated cubic spline fits to the kernel density estimates. Pertsemlidis et al. used an exponential of a Fourier series to calculate log probability densities of φ,ψ data [18]. Hovmöller et al. produced smoothed Ramachandran distributions for all 20 amino acids, and differentiated among different secondary structures; however, the manner of smoothing was not described [19]. Dahl et al. [12] and Lennox et al. [13] used Dirichlet process mixture models to obtain Ramachandran distributions for all 20 amino acids. The Dirichlet process approach is similar to kernel density estimation in that it yields an overall density estimate that is a superposition of component density functions, but the component densities are not located at the data points, and the number of component densities is unknown and inferred from the data [20]. This latter fact places the Dirichlet process approach in the general class of so-called Bayesian nonparametric methods [21]. Here, “nonparametric” does not mean an absence of parameters, but rather means that the number of parameters is not fixed in advance and can grow as data accrue.

Ramachandran distributions may also be affected by the identity or conformation of neighboring amino acids. In particular, it has long been known that residues that precede proline have quite different Ramachandran distributions [22], with significantly less density in the α and left-handed regions of the Ramachandran map. They also exhibit additional density in the so-called ζ region [23], near φ,ψ = (−130°, +80°), due to favorable van der Waals and electrostatic interactions [2]. The effect of local sequence on backbone conformation initially was used for the purpose of secondary structure prediction [24], [25]. A number of groups have discussed the effect of local sequence, usually plus or minus one amino acid, on backbone conformational distributions [15], [26], [27], [28], [29], [30], [31]. This has been examined as a violation of the Flory isolated pair hypothesis, which states that conformations of individual dihedral angle pairs in a polymer are approximately independent of the conformations and/or residue identity of their neighbors [32]. Pappu et al. demonstrated by enumerating conformations of polyalanine that this is not true for neighboring conformations in peptides [29]. Zaman et al. used molecular dynamics simulations of monomers, dimers, and trimers to determine the nearest-neighbor effects of conformation and amino acid type on backbone conformations and entropy [27]. From the same group, Jha et al. examined experimentally determined distributions in coiled regions in a set of 2020 proteins of better than 2.0 Å resolution and found strong neighbor dependence on the populations in the α, β, and Polyproline II (PPII) regions of the Ramachandran map [26]. Erman et al. also examined neighbor residue-type dependence of which regions (α, β, etc.) were populated as a method for predicting these regions given local sequence context from a statistical mechanical theory [30], [31]. Betancourt and Skolnick used 7070 chains from the PDB to determine the conformational properties of triplets of amino acids, in terms of occupied basins of the Ramachandran map, as well as other distributions such as the pair 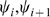 [28]. They used the data to produce a low-resolution potential energy function for backbone conformations that depends on local sequence. Lennox et al. [13] found that smooth density estimates for 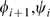 pairs, which span a pair of residues and thus capture a limited form of neighbor dependence, yield better estimates of the Ramachandran distribution than those based on standard 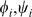 pairs.

A limitation of efforts to capture neighbor dependence is that the data become fractionated into groups that may contain small numbers of data points. This can yield inaccurate estimates of the densities, defeating the purpose of separating the data into groups. This problem is compounded if we also wish to separate data by secondary structure, or by any of a variety of other contextual variables. Our approach to addressing this general problem is to make use of the concept of a *hierarchical Bayesian model*. A hierarchical model is akin to a phylogeny, where the models for individual groups of data are at the leaves, and models are related if they are nearby each other in the tree. Specifically, we make use of a recent development in Bayesian nonparametric statistics known as the *hierarchical Dirichlet process* (HDP) [33]. In the HDP approach, as we discuss in the Methods section, evidence for a region of high density in one group of data can be transferred to a related group. In particular, we can use the HDP to tie together the density estimates for a given residue with different right or left neighbor residue types. This approach allows us to exploit commonalities among these densities so as to combat the data sparsity problem while allowing the individual densities to exhibit idiosyncratic characteristics.

In this paper, we determine both neighbor-independent and neighbor-dependent Ramachandran distributions for all 20 trans amino acids as well as cis proline (21 distributions) and for all 420 left and 420 right-neighbor-amino acid type pairs. We use a set of 3038 proteins at resolution of 1.7 Å or better and use electron density calculations to remove residues that are not well-fit to the density [34]. We explore the features of different input data sets, for instance including or excluding 3_10_-helix and turn residues from longer loop regions.

We examine some clear trends in these distributions. These include not only the influence of neighboring proline residues, but also aromatics, β-branched residues, hydrogen-bonding residues, and glycine. Our primary purpose for developing these potentials is to improve protein structure prediction. We perform a number of tests including loop modeling with Rosetta [8] as well as prediction of φ,ψ values of loops residues purely from local sequence. The neighbor-dependent distributions provide better results in both cases.

The distributions are available for download from http://dunbrack.fccc.edu/hdp.

## Results

### Characteristics of the data set

The data set in this paper consisted of 3038 proteins with available electron densities from the Uppsala Electron Density Server [35]. After removing residues with electron density in the bottom 20th percentile and restricting the set to loop residues with no missing backbone atoms and at least three residues away from α helix (H) or β sheet (E), as identified with the program Stride [36], we obtained a set of 62,345 residues (the TCBIG set, for Stride one-letter designations of Turn, Coil, β-Bridge, π-Helix, 3_10_-Helix respectively). We created a second set by removing the “regular” secondary structures of 3_10_-helices and π-helices and those residues that neighbor them. The result is a set of 44,112 residues (the TCB set). In both sets, we kept so-called “Bridge” residues, since these sometimes occur in long loop regions as backbone-backbone hydrogen bonds. The percentages in each secondary structure for each set are given in Table 1. The percentages in each Ramachandran region for each set are given in Table 2. The regions are defined in Methods, and consist of A (α helix region), B (β sheet regions), P (polyproline II region), L (left-handed helix), and E (ε or extended region, the lower right and upper right regions of the φ,ψ map, accessible primarily to glycine). Cis residues are counted separately.

**Table 1:** Ramachandran and secondary structure assignments for data set (in percent).

	TCBIG	TCB
**Count**	62345	44112
**Turn**	50.1	62.4
**Coil**	29.4	31.6
**3_10_ helix**	15.4	-
**Bridge**	5.0	6.0
**π helix**	0.1	-

TCBIG includes Turn, Coil, Bridge, π-helix, and 3_10_-helix residues.

TCB includes Turn, Coil, and Bridge residues.

**Table 2:** Ramachandran totals for each set and for individual Stride assignments for the TCBIG set (in percent).

	TCBIG	TCB	Turn	Coil	3_10_ helix	Bridge	π-helix
**A**	41.1	31.9	42.4	17.1	95.4	0.7	85.9
**B**	22.0	25.8	18.5	32.7	0.1	63.0	2.8
**P**	26.3	30.2	24.1	42.1	0.9	33.1	2.8
**L**	7.5	8.4	11.4	4.6	2.7	0.3	5.6
**E**	2.5	2.8	2.4	3.3	1.1	2.9	0.0
**cis**	0.7	0.9	1.2	0.3	0.0	0.04	2.8

A: −200°≤φ<0°, −120°<ψ≤40°, 90°≤ω≤270°.

B: −90°≤φ<0°, 40°<ψ≤240°, 90°≤ω≤270°.

P: −200°≤φ<−90°, 40°<ψ≤240°, 90°≤ω≤270°.

L: 0°≤φ<160°, −90°<ψ≤110°, 90°≤ω≤270°.

E: 0°≤φ<160°, 110°<ψ≤270°, 90°≤ω≤270°.

cis: −90°≤ω<90°.

Removing the regular secondary structures, 3_10_-helix and π-helix, from TCBIG has a large effect on both the Ramachandran distributions and contributions of turns and coil. TCBIG is 41% α while TCB is 32%. TCBIG is 50% Turn while TCB is 62% Turn. Turns contribute substantially to long loops, and also to the population in the Ramachandran α region. Finally, we also performed calculations on Turn residues alone and Coil residue alone, making the T and C sets respectively. These are relatively small sets of 27,532 and 13,945 residues. The neighbor-independent distributions are likely to be reasonable, but the neighbor-dependent ones may require larger data sets with lower resolution and/or less stringent cutoffs for electron density or mutual sequence identity.

### Neighbor-independent Ramachandran distributions

**Figure 1:**
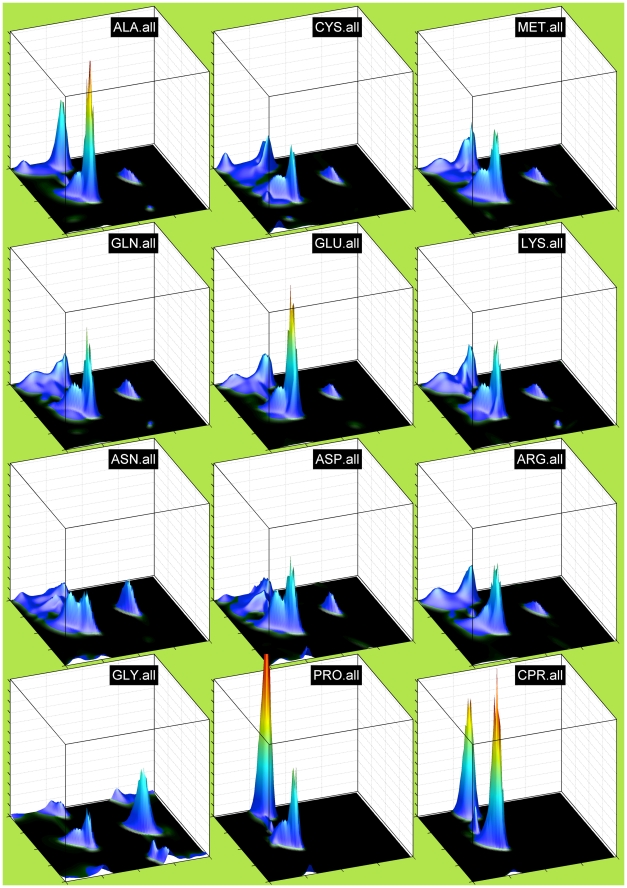
Neighbor-independent Ramachandran distributions (probability densities) for 12 amino acid types including cis proline (CPR) from the HDP simulation (see Figure 2 for remaining amino acid types). The axes are as follows: x-axis (horizontal) = φ from −180° to 180° with gridlines and ticks at −180°, −90°, 0°, +90°, and +180°. y-axis = ψ from −180° to 180°. z-axis (vertical) = probability density functions. All plots are scaled to a common maximum probability height, the third highest peak among all plots. Thus, Gly and Pro extend beyond the plotted vertical axis.

**Figure 2:**
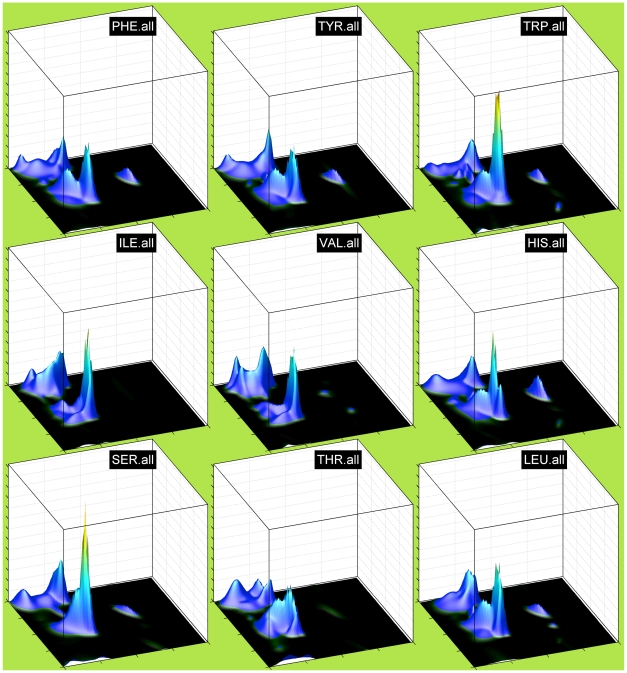
Neighbor-independent Ramachandran distributions for remaining 9 amino acid types (continued from Figure 1). For axis information, see caption to Figure 1.

**Figure 3:**
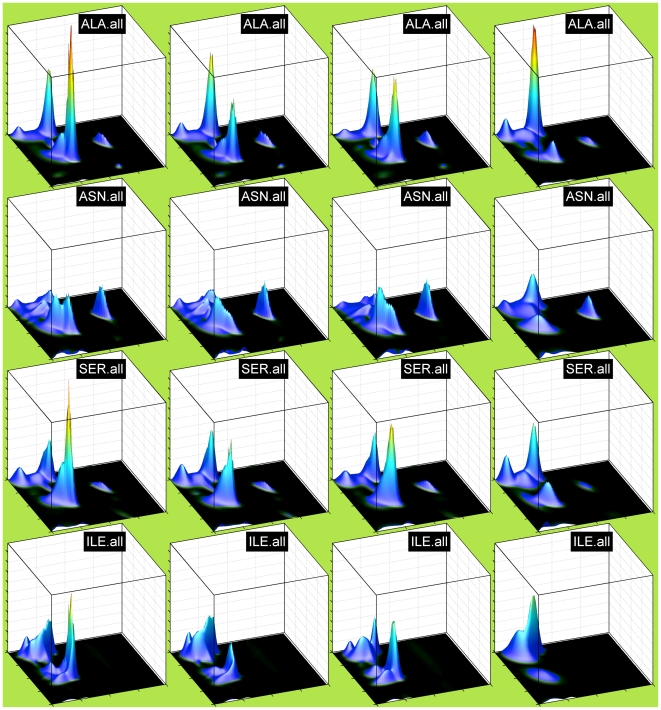
Comparison of different data sets for neighbor-independent Ramachandran distributions for 4 amino acid types. Rows (from top to bottom): Lys, Met, Leu, Glu. Columns (from left to right): TCBIG, TCB, Tonly, Conly. All plots are scaled to a common maximum probability height.

In Figures 1 and 2, we show neighbor-independent Bayesian nonparametric density estimates of the Ramachandran distributions of all 20 amino acids for the TCBIG set with cis and trans proline plotted separately. These are smoother than many previous Ramachandran distributions and show the differences among the 20 amino acids clearly (the jagged appearance at the top of peaks is an artifact of plotting the surface with polygons). In this and subsequent figures, the notation XXX.yyy or yyy.XXX means the probability density estimate for residue XXX with yyy as right or left neighbor respectively. The residue types, even outside of Gly and Pro, have quite distinct Ramachandran distributions. Because β turns are a large proportion of both the TCBIG and TCB data sets, we also calculated smooth density estimates of the Ramachandran distributions of turn and coil residues separately (Stride designations T and C), and in Figure 3 we plot side-by-side the Ramachandran distributions for TCBIG, TCB, T-only, and C-only for Ala, Asn, Glu, and Ile. The TCB set loses the sharp peak near (φ,ψ) = (−50°,−25°) which is a result of the 15% of residues in the TCBIG set that are in 3_10_-helix. As noted by others [26], coil and turn residues (columns 3 and 4 respectively) have quite different distributions with the turn set having higher α content and the coil set having higher polyproline-II content.

We calculated the Hellinger distance (see Methods) between all residue types, and the values for a subset of 12 residues are given in Table 3 for TCBIG. The table shows the calculated Hellinger distances times 100, and we refer to these values in what follows as “the Hellinger distance,” which will be a value between 0 for identical probability densities and 100 for completely non-overlapping densities. Very similar residue pairs such as Phe/Tyr and Val/Ile have Hellinger distances of about 8 or 9. Very different distributions such as any residue with Gly or Pro have Hellinger distances in the range of 40 to 60. Outside of Gly and Pro, most distances are in the range 10 to 30. Alanine is *not* a typical residue; most side chains with a single γ heavy atom have smaller Hellinger distances to each other than they do to Ala.

**Table 3:** Hellinger distances for neighbor-independent distributions in the TCBIG set.

	PHE	TYR	GLN	LYS	VAL	ILE	ALA	ASN	PRO	GLY
**PHE**		8	10	12	21	22	19	19	44	46
**TYR**	8		11	12	21	21	19	19	44	46
**GLN**	10	11		9	21	22	16	18	43	45
**LYS**	12	12	9		20	20	16	22	42	45
**VAL**	21	21	21	20		9	28	32	47	52
**ILE**	22	21	22	20	9		16	32	47	52
**ALA**	19	19	16	16	28	16		25	34	44
**ASN**	19	19	18	22	32	32	25		46	43
**PRO**	44	44	43	42	47	47	34	46		53
**GLY**	46	46	45	45	52	52	44	43	53	

Values are 100× Hellinger distance and rounded to the nearest integer.

Between distributions derived from the TCBIG and TCB sets, the Hellinger distances of the same residues in each set (e.g., Ala in TCBIG vs. Ala in TCB) range from 5 to 11, with the larger values coming from hydrophobic residues, which are underrepresented in 3_10_-helix. Comparison of turns with TCB or TCBIG for a single residue type produces Hellinger distances in the range of 9 to 14; these data sets are 50 and 62% turn respectively. On the other hand, coil distributions are quite different from the TCB and TCBIG sets with Hellinger distances in the range of 14 to 22 (data not shown).

### Neighbor-dependent Ramachandran distributions

**Figure 4:**
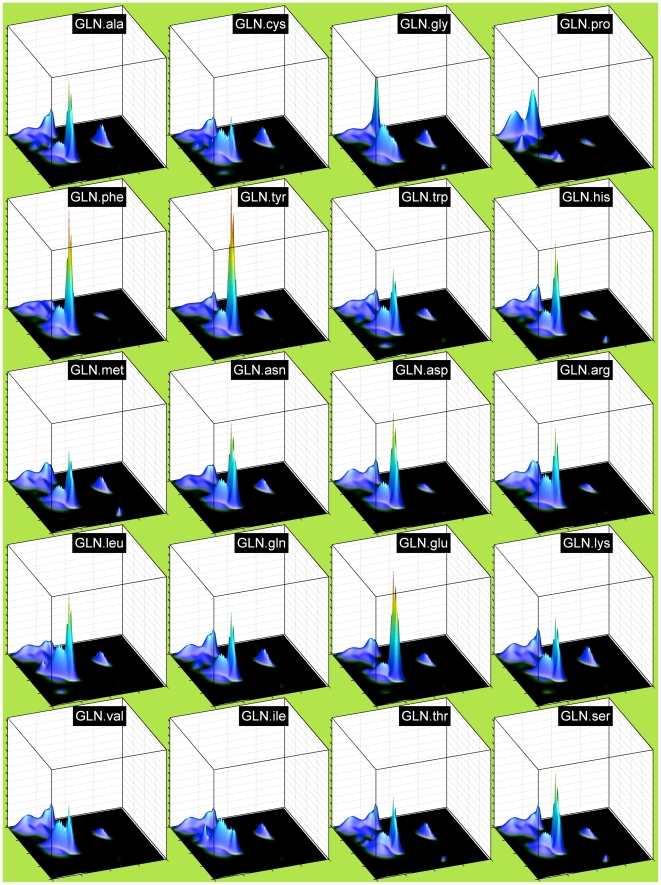
Right neighbor-dependent Ramachandran distributions of glutamine with each of 20 amino acid types as right neighbors. All plots are scaled to a common maximum probability height.

In Figure 4, we show the effect of all 20 possible right neighbors on the Ramachandran distribution of Gln. Gln behaves typically in terms of neighbor effects. Certain neighbor types have consistent effects in terms of increasing or decreasing the α, β, and/or PPII regions for most central residue types, and the residues are grouped accordingly in the figure. Pro on the right exerts the largest effect and this has been well-studied previously [2], [22], but with these calculations we provide smooth, statistically reasonable pre-Pro Ramachandran distributions for all 20 amino acids. The GLN.pro map shows the features typical of pre-Pro distributions – very low α (A) population, lower L population than non-pre-Pro distributions, and the so-called ζ conformation [2], [23], which is a bump just below the β (B) region at φ,ψ = (−130°, +80°).

**Figure 5:**
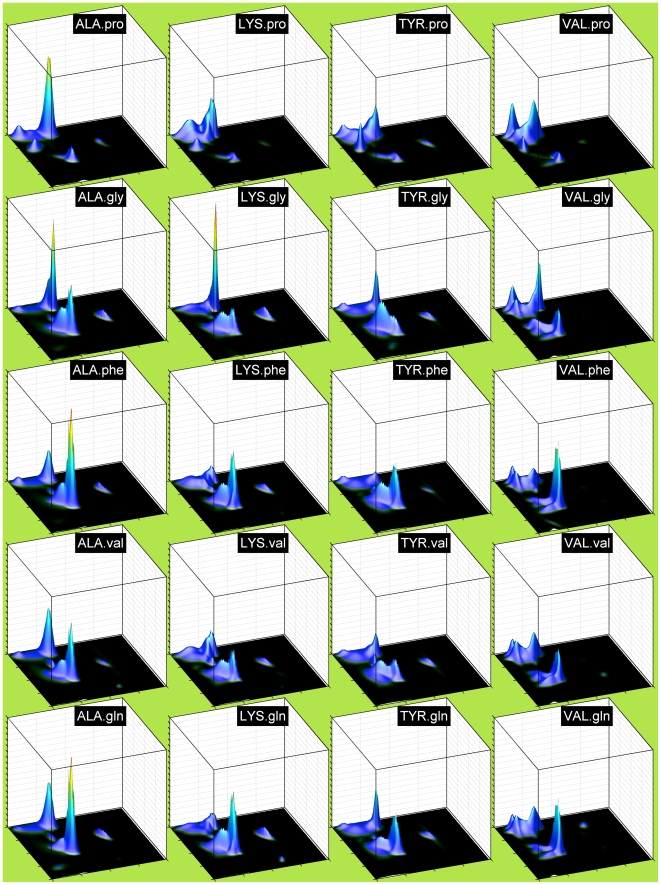
Right Neighbor-dependent Ramachandran distributions of four residues with different right neighbors. Columns (left to right): central residues ALA, LYS, TYR, VAL. Rows (top to bottom): right neighbors pro, gly, val, gln. All plots are scaled to a common maximum probability height.

**Figure 6:**
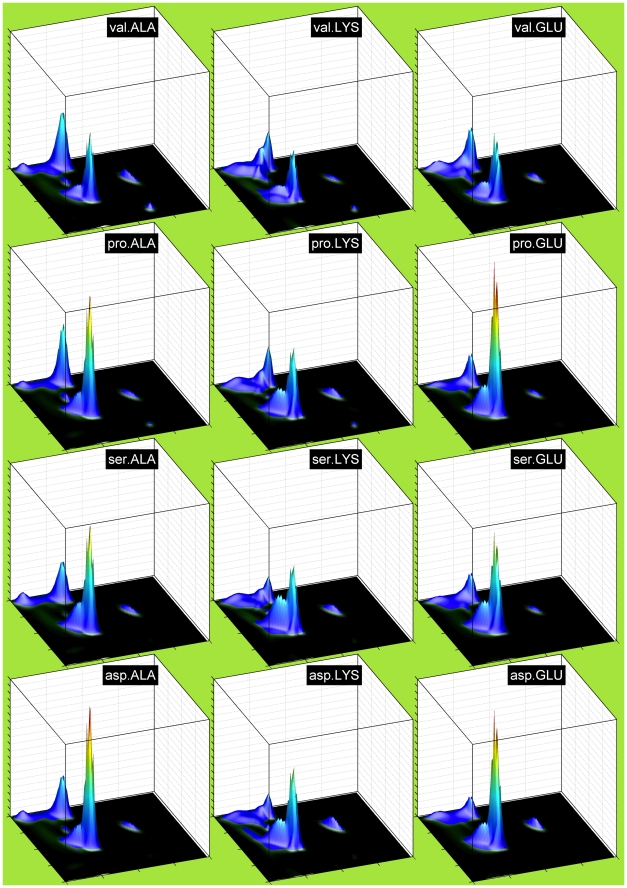
Left neighbor-dependent Ramachandran distributions of three residue types. Columns (left to right): central residues ALA, LYS, and GLU. Rows (top to bottom): left neighbors val, pro, ser, and asp. Vertical scale is the same as Figure 5.

Other groups of residues also have particular effects as neighbors. Aromatic residues as right neighbors, especially Phe and Tyr, suppress the P region and increase the A region. Val and Ile suppress A in favor of broadly distributed B and P density, while Gly strongly favors P, most likely due to an increase in Type II turns. Type II turns consist of residue 2 (of 4 residues in the turn) in a P conformation and residue 3 in an L conformation, most accessible to glycine. Negatively charged residues also seem to increase A. To show that these are general effects, in Figure 5 we show Ramachandran plots for ALA, LYS, TYR, and VAL with right neighbors equal to pro, gly, phe, val, and gln. Gln behaves as a relatively neutral neighbor. The other neighbor types have similar effects on these residues as they do on Gln shown in the previous figure. In Figure 6 we show the effects of left neighbors. Val and Ile tend to reduce A conformations while Pro, Ser, and Asp tend to increase A conformations.

**Figure 7:**
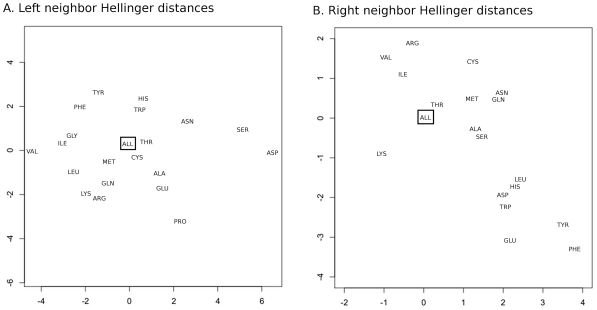
Multi-dimensional scaling plots of average Hellinger distances. **A**: left neighbors. **B**: right neighbors. The distance between any two neighboring residue in the plots is approximate. “ALL” indicates all residues as neighbor and is boxed in each figure.

To quantify the effects, Table 4 contains the Hellinger distances for right neighbors of Gln, which shows the similar behaviors of Phe and Tyr, Val, and Ile, and the different behaviors of Gly and Pro as neighbors. Ile and Val as right neighbors of Gln are not as similar as they are for most other amino acid types, where the average Hellinger distance is about 7. We calculated the average Hellinger distances between each pair of neighbors over all the central amino acid types, e.g., for right neighbors *R_i_* and *R_j_*:
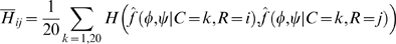
and then used classical multi-dimensional scaling [37] to plot these distances approximately in two dimensions. The results are shown in Figure 7 for both left neighbors (Figure 7A) and right neighbors (Figure 7B). For the right neighbors, we omitted Pro and Gly from the graph since they lie far from the others. Pro is at coordinate (−25.5, −1.3) and Gly is at (0.7, 11.2). Residues with similar properties are mostly grouped together, e.g. Val and Ile, Phe and Tyr, Lys and Arg (as left neighbors), Asn and Gln (as right neighbors), etc. The distances are on relatively similar scales with Val and Asp having the largest Hellinger distance at 11.7 for left neighbors, and Ile and Glu at 12.6 for the right neighbors (excluding Gly and Pro as right neighbors).

**Table 4:** Hellinger distances for right-neighbor-dependent distributions of glutamine in the TCBIG set.

	PHE	TYR	GLN	LYS	VAL	ILE	ALA	ASN	PRO	GLY
**PHE**		8	15	16	14	17	15	12	35	20
**TYR**	8		13	14	15	18	12	8	35	19
**GLN**	15	13		9	9	11	7	9	27	12
**LYS**	16	14	9		9	12	8	9	27	14
**VAL**	14	15	9	9		10	12	11	27	12
**ILE**	17	18	11	12	10		13	13	29	12
**ALA**	15	12	7	8	12	13		9	27	15
**ASN**	12	8	9	9	11	13	9		32	15
**PRO**	35	35	27	24	27	29	27	32		32
**GLY**	20	19	12	14	12	12	15	15	32	

Values are 100× Hellinger distance and rounded to the nearest integer.

Residues listed are right neighbors of glutamine.

### Loop prediction with neighbor-dependent Ramachandran distributions

**Figure 8:**
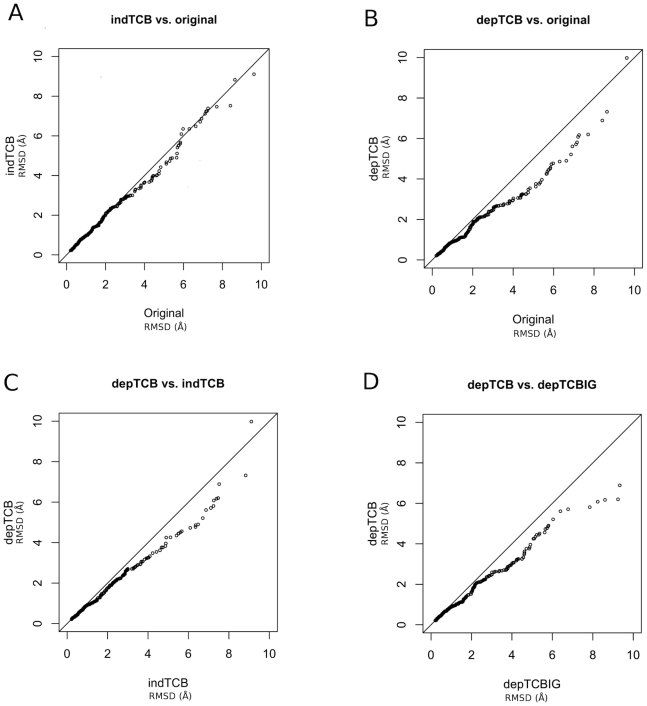
Quantile-quantile (QQ) plots for 230 loop predictions by Rosetta using different Ramchandran distributions. Each figure plots the numerically sorted Cα RMSDs for predictions from one form of Ramachandran distributions against the numerically sorted Cα RMSDs from another. A: neighbor-independent TCB distributions vs. original distributions in Rosetta; B: neighbor-dependent TCB distributions vs Rosetta original distributions; C: neighbor-dependent TCB distributions vs neighbor-independent TCB distributions; D: neighbor-dependent TCB distributions vs neighbor-dependent TCBIG distributions.

To test whether the neighbor-dependent Ramachandran distributions will have utility in protein structure prediction, we ran a benchmark of loop predictions developed by Soto et al. [38], consisting of 290 loops from length 8 to length 13. Rosetta was used to predict the structures of these loops in the context of the rest of each experimental protein structure [39]. The goal was not to judge the accuracy of Rosetta but to compare the different Ramachandran distributions for scoring and energy minimization. The results are shown in Figure 8 in the form of Q-Q plots (quantile-quantile) [40]. To produce a Q-Q plot, each group of data are sorted numerically, and the resulting vectors are plotted against one each other. Note that the x and y axes are RMSDs rather than 1/290^th^ quantiles. Significant deviations from the line y = x indicate differences in the distribution of the two data sets.

In Figure 8A, loop prediction Cα RMSDs using the neighbor-independent TCB Ramachandran distributions are plotted against loop predictions using the Ramachandran distributions currently in Rosetta (“original”) [8]. The plot shows a small benefit to the new Ramachandran distributions in the 3–6 Å range of RMSD. The TCBIG distributions show similar results (not shown). In Figure 8B, the neighbor-dependent distributions are compared to the original distributions and exhibit much larger differences down to 2.0 Å in RMSD. To compare the neighbor-dependent and neighbor-independent distributions from the TCB set, the Q-Q plot is shown in Figure 8C. Again, the neighbor-dependent distributions have an advantage over the neighbor-independent distributions.

Finally, in Figure 8D the TCB set is shown to produce better predictions than the TCBIG set, which emphasizes the need to choose the right Ramachandran distributions depending on the prediction task. To explore why, we evaluated the Stride assignments and the Ramachandran distributions for the benchmark loops. The benchmark is 59% Turn, 36% Coil, 3% Bridge, and only 1.5% 3_10_ Helix. As shown in Table 1, this distribution matches the TCB (62% Turn) set much more closely than the TCBIG set (50% Turn). The benchmark is 31% α helical region and 57% β and Polyproline II region. This is much closer to the TCB set (32% A and 56% B+P) than the TCBIG set (41% A and 48% B+P), as shown in Table 2.

As a test independent of loop sampling methodology and scoring functions, we took a set of 2,074 proteins with resolution better than 1.8 Å, and for loops longer than 8 residues, we predicted the φ,ψ value by selecting the grid point with the largest probability:
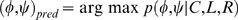
where C is the central residues whose φ,ψ are being predicted and L and R are the identities of its left and right neighbors. We then compared these predictions with the values from the crystallographic structure. The results are shown in Table 5, which shows the percentage of residues whose φ or ψ are predicted within 40° and also the mean absolute deviation in φ and ψ between the native and predicted values. The improvements from the neighbor-independent distributions to the neighbor-dependent distributions are evident. For instance for Leu, the neighbor-independent distributions are able to predict the ψ values for only 37.3% of residues within 40°, while the neighbor-dependent distributions predict 52.4% correct, for an improvement of 15.1% of all leucines. This is a 40% improvement of the neighbor-independent rate (15.1/37.3). Dihedral φ is concentrated in a smaller region than ψ and so is harder to improve than ψ. The largest improvements are for hydrophobic residues, while residues that show different behaviors in turns vs, coils (Gly, Pro, Asn) show less improvement, probably because the neighbor effects are context-dependent (Turn or Coil).

**Table 5:** Ramachandran predictions for maximum probability of neighbor-dependent vs. neighbor independent distributions for loop residues in 2074 proteins.

Res	Count	%	%imp	%	%imp	Ave.	° imp	Ave.	° Imp.
		|Δφ|<40°		|Δψ|<40°		|Δφ|		|Δψ|	
ASN	11686	55.9	−10.6	49.3	3.2	46	−10	62	6
GLY	24367	64.2	−1.2	59.0	1.4	58	−0	64	6
SER	13998	68.1	−0.6	46.3	5.7	41	−1	89	9
PRO	15820	99.5	−0.1	66.3	6.0	60	−0	60	10
LYS	10429	63.9	−2.9	49.8	5.8	44	−1	75	11
TRP	2613	64.9	1.0	48.1	6.2	42	1	81	10
THR	11529	60.0	−2.4	47.3	6.3	41	−2	86	12
GLU	10678	74.4	−0.9	57.5	7.8	50	−0	67	12
ARG	8449	61.3	−1.7	48.7	7.8	41	−1	77	13
GLN	6369	65.6	0.7	47.0	5.0	42	0	75	12
ASP	15847	72.4	4.0	49.3	8.7	41	2	69	13
HIS	4922	56.1	0.6	44.4	9.1	35	0	78	15
TYR	5908	60.0	1.9	44.2	7.8	36	1	80	16
CYS	2643	60.0	0.2	43.4	11.1	32	0	83	17
MET	2504	67.8	−0.8	49.3	15.3	34	−1	78	23
PHE	6256	58.6	6.0	44.0	12.8	31	4	78	23
LEU	11875	74.1	4.9	52.4	15.1	37	3	75	24
ILE	6402	61.4	5.1	58.1	28.8	29	3	68	42
VAL	8552	61.3	5.5	57.2	28.9	28	4	71	43

Δφ<40° and Δψ<40° indicate predicted φ,ψ position with maximum probability is less than 40 degrees away in φ and ψ respectively from native position calculated with the neighbor-dependent distributions. %imp is the increase in the percent of all residues of each type that are predicted correctly with the neighbor-dependent distributions compared to the neighbor independent distributions. Ave. |Δφ| and |Δψ| are the mean absolute deviations of the most probable φ,ψ values from the native values of φ and ψ calculated with the neighbor-dependent distributions. Deg. Imp indicates how much better predictions are (that is lower) with the neighbor-dependent distributions than with the neighbor-independent distributions.

### Interaction of neighboring side chains with the protein backbone

It is of some interest to understand the origin of the neighbor-dependent backbone conformation propensities observed in this work and in similar analyses that have appeared previously. The analysis is complicated by the preponderance of β turns in the two data sets. Such turns are defined by Cα-Cα distances of less than 7 Å between residue 1 and residue 4 in a four-residue segment. β turns are categorized by the conformations of residues 2 and 3, and follow some well-studied amino acid preferences at all four positions. [41]

To determine whether the neighbor effects were dependent on whether residues were in turns or in coil regions of loops, we used the raw data to determine the percentages of residues in A, B, and P regions of the Ramachandran map depending on the neighbor residue types for coil, turns, and all residues in the TCB set. The results are presented in Table 6. Note, these are exact values from raw counts of the data, not from the probability densities that result from them.

**Table 6:** Changes in Ramachandran regions for right and left nearest neighbors in Coil, Turn, and All loop residues.

		A			B			P	
RIGHT neigh.	Coil	Turn	TCB	Coil	Turn	TCB	Coil	Turn	TCB
%Rama	19.4	46.4	35.2	32.1	15.2	23.3	40.3	22.2	28.6
**ILE**	***−7.3***	***−4.2***	***−8.2***	1.4	2.4	2.7	4.9	1.1	5.5
**VAL**	***−7.8***	***−4.9***	***−7.4***	***−1.2***	2.5	1.7	9.5	0.2	5.2
**LYS**	***−3.1***	***−9.5***	***−6.8***	***−6.0***	4.0	1.0	8.2	3.4	4.0
**MET**	***−0.2***	***−5.6***	***−4.3***	***−0.8***	1.6	0.3	***−1.3***	0.6	1.5
**LEU**	***−7.7***	1.6	***−3.8***	0.5	***−0.8***	0.8	7.1	***−2.1***	2.9
**ARG**	***−0.7***	***−5.0***	***−3.4***	***−7.7***	0.4	***−2.8***	7.3	1.0	3.7
**GLN**	***−4.9***	***−3.5***	***−3.1***	***−5.5***	0.2	***−1.3***	10.3	0.8	2.6
**ALA**	***−2.0***	***−1.4***	***−2.2***	1.4	2.6	2.6	1.9	***−1.0***	0.5
**THR**	***−2.4***	0.5	***−0.6***	***−2.9***	***−0.6***	−1.2	3.0	***−2.7***	***−0.6***
**CYS**	1.4	2.9	0.1	***−6.6***	***−5.7***	−4.6	0.4	***−5.9***	***−1.4***
**SER**	3.8	0.5	1.6	***−2.6***	1.0	0.1	0.1	***−2.8***	***−2.0***
**GLU**	2.3	0.0	2.0	1.1	2.9	1.7	***−1.6***	***−0.1***	***−1.7***
**TRP**	***−0.7***	5.1	3.1	10.3	1.6	4.7	***−10.1***	***−7.3***	***−8.5***
**GLY**	14.0	***−3.6***	3.2	***−1.7***	***−4.6***	***−4.7***	***−10.6***	11.4	3.6
**ASN**	2.8	2.5	3.9	1.3	***−2.0***	***−1.8***	***−5.8***	***−0.6***	***−3.0***
**PHE**	***−0.9***	7.8	4.4	10.8	***−0.6***	3.0	***−10.0***	***−6.0***	***−6.6***
**TYR**	3.5	7.3	5.3	8.0	0.2	2.8	***−10.5***	***−6.3***	***−7.1***
**ASP**	1.0	7.0	5.9	3.6	0.2	1.2	***−3.3***	***−2.6***	***−4.0***
**HIS**	1.6	7.4	6.4	1.8	***−1.2***	***−0.7***	***−6.7***	***−4.7***	***−5.9***
**PRO**	***−16.8***	***−40.3***	***−30.6***	9.5	31.1	22.6	11.4	18.4	15.2
**LEFT neigh.**									
**ILE**	***−3.4***	***−13.7***	***−10.7***	***−1.2***	8.2	4.9	5.9	9.4	8.9
**VAL**	***−4.0***	***−11.6***	***−10.0***	0.4	3.6	3.4	7.5	8.9	9.3
**LEU**	***−4.1***	***−9.6***	***−8.1***	***−0.9***	0.8	1.3	5.4	11.0	8.4
**LYS**	***−10.6***	***−4.0***	***−7.8***	3.3	***−0.7***	1.9	10.1	2.5	6.3
**PHE**	***−1.7***	***−12.1***	***−7.8***	9.1	4.8	5.7	***−5.4***	4.3	0.6
**GLN**	***−4.9***	***−8.2***	***−7.5***	1.4	2.3	2.4	5.6	2.4	4.1
**MET**	***−4.3***	***−5.5***	***−5.9***	***−4.8***	***−1.9***	***−2.9***	6.8	3.1	5.9
**TYR**	2.9	***−9.4***	***−4.8***	7.7	3.8	4.6	−9.6	2.0	***−1.9***
**ARG**	***−4.3***	***−3.5***	***−4.7***	−1.2	1.7	1.3	7.4	3.5	5.5
**GLU**	***−7.8***	***−1.6***	***−3.2***	***−0.2***	***−0.3***	***−1.5***	7.1	***−1.2***	2.3
**TRP**	***−1.5***	***−4.8***	***−2.6***	4.8	5.8	5.2	***−3.9***	***−3.6***	***−4.8***
**ALA**	−4.6	1.0	−1.1	1.9	−1.0	0.7	2.5	***−0.7***	0.0
**GLY**	3.4	***−0.1***	***−0.2***	−0.1	2.8	2.6	***−3.2***	3.4	1.6
**HIS**	1.7	***−1.3***	0.1	4.3	2.3	2.6	***−6.4***	***−0.8***	***−3.0***
**THR**	4.4	***−0.1***	1.3	***−5.1***	***−1.9***	***−3.1***	***−2.7***	0.7	***−0.1***
**CYS**	14.1	***−4.2***	2.0	***−7.8***	***−1.4***	***−3.8***	***−11.1***	1.6	***−2.6***
**PRO**	***−4.3***	8.4	4.4	1.1	***−4.8***	***−2.7***	4.7	***−3.9***	***−1.5***
**ASN**	12.9	6.6	9.2	***−1.4***	***−0.4***	***−1.6***	***−13.1***	***−6.0***	***−8.2***
**SER**	13.2	10.9	12.2	***−2.9***	***−3.5***	***−4.1***	***−12.0***	***−6.8***	***−8.7***
**ASP**	6.9	12.9	14.8	***−6.3***	***−4.1***	***−7.0***	***−6.2***	***−10.4***	***−11.2***

The first row is the percent in each region for each secondary structure type (Stride C, T, or T+C+B). Top: right neighbors. Bottom: left neighbors. Each cell gives the difference as a percentage of all residues in the secondary structure type. E.g., 40.3% of non-pre-Pro Coil residues are in a P conformation, while 45.2% (40.3+4.9) of non-pre-Pro Coil residues with Ile as right neighbor are in a P conformation. Negative numbers are in bold italic type.

The first row of numbers gives the percentage in A, B, or P of all Coil, Turn, or TCB non-pre-Pro residues. That is, 19.4% of Coil non-pre-Pro residues are in A conformation. We exclude pre-Pro residues, due to the large effect Pro has when it is a right neighbor. The numbers in the top half of the table show the effect of the right neighbors listed in the first column as changes in the percentage of all residues in each secondary structure type (C, T, or TCB). That is, while 19.4% of non-pre-Pro Coil residues are in A, only 12.1% of pre-Ile residues in Coil are in A. The pre-Pro numbers in the table are relative to all residues in each group (C, T, or TCB).

Some effects are seen in both Turn and Coil conformations, while others are Turn or Coil specific. So for instance, Ile and Val neighbors to the right decrease A populations for both Turn and Coil, although for Coil the P population rises more than the B population compared to Turn. Aromatic residues to the right decrease P for both Turn and Coil, although in this case for Turn A goes up while for Coil B goes up. Asn, Ser, and Asp to the left decrease P and to a lesser extent B and increase A significantly both in Turn and in Coil. Ile and Val to the left decrease A and increase P for both Turn and Coil, while also increasing B for Turn, probably due to turns of type β_ε_γ_L_ (residues 2 and 3) according to the nomenclature of Wilmot and Thornton [41]. This is a form of distorted type II turns, which prefer Val and Ile at position 1, and hence B and P at position 2. Some effects are completely conformation specific. Gly to the right increases P and decreases A in Turn conformations (due to Type II turns with Gly at position 3), while having the opposite effect on Coil. Pro to the left increases A and decreases B and P for Turn, probably due to an increase in Type I turns with Pro at position 2, while having exactly the opposite effects for Coil conformations.

**Figure 9:**
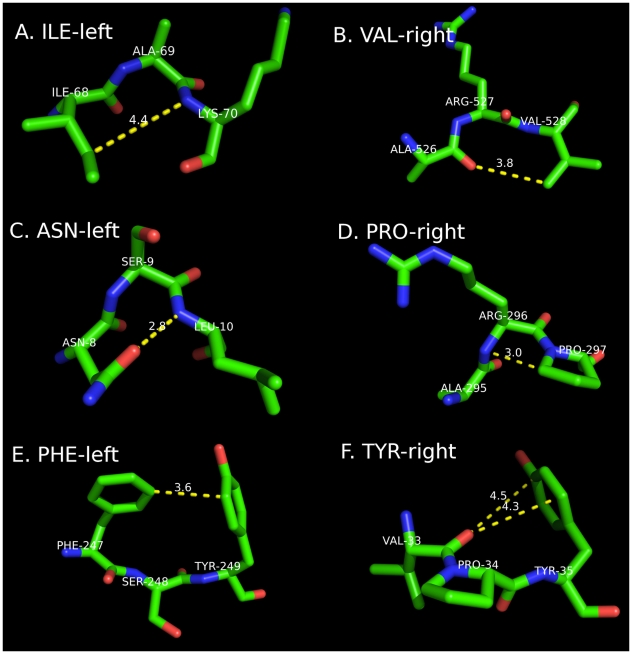
Neighbor-dependent effects. **A**. ILE (left) – ALA (center conformation A, coil, PDB entry 1N55, residue 69 of chain A). Ile likely prevents H-bond acceptor to N_i+1_. **B**. VAL (right) – ARG (cent, A, coil, 2FWH_527A). Val likely prevents H-bond donor to O_i−1_. **C**. ASN (left) – SER (cent, A, coil, 3C8W_9A). Asn forms hydrogen bond to N_i+1_. **D**. PRO (right) – ARG (cent, A, coil, 2RKV_296A). Very close steric bump of Pro CD with N*_i_*. **E**. PHE (left) – SER (cent, B, coil, 2GS8_248A). Favorable hydrophobic interaction with Tyr*_i_*_+1_. **F**. TYR (right) – PRO (cent, P, turn, 3CA8_34A). Tyr unfavorable electro. interaction with O_i−1_.

We investigated some of these preferences visually in both turns and coils in order to identify potential favorable or unfavorable interactions that may be responsible for the observed propensities listed in Table 6. Several of these interactions are shown in Figure 9 in images of residue triples, chosen to demonstrate the interaction of the left or right neighbor side chain with the other two amino acids of the tripeptide depicted.

Ile as a left neighbor to an A conformation in Coil is shown in Figure 9A. In this conformation, the β branched amino acid blocks access of hydrogen-bond acceptors to the NH of the amino acid to the right of the central residue (*i*), whose relative position is determined by φ, ψ of residue *i* and ψ of residue *i*−1. In this image, atom Cγ_1_ of Ile is 4.4 Å from N*_i_*_+1_. A residue with only a single Xγ atom might have Xγ in the position of Cγ_2_ in this residue, thereby allowing access to NH*_i_*_+1_. This interaction occurs when residue *i*−1 is in a B conformation, which is more common for Coil than for Turn. A similar effect is shown for Val to the right in Figure 9B, with a close contact of Cγ_2_ with backbone atom O*_i_*_−1_.

Polar interactions and side-chain/backbone hydrogen bonds are also likely responsible for some of the observed trends in Table 6. In Figure 9C, an Asn Oδ_1_ at *i*−1 acts as a hydrogen bond acceptor to NH*_i_*_+1_ and thus in fact favors an A conformation at position *i*, both in Turn and in Coil (as shown). Asp Oδ_1_ Oδ_2_ at *i*−1 can make the same hydrogen bond with NH*_i_*_+1_, and the population of residue *i* is also increased in A conformations (Table 6). Ser Oγ behaves similarly (not shown). It is interesting to note that these three residues, Asn, Asp, and Ser, cluster together in the two-dimensional projection of their distances as left neighbors (Figure 7A) but not as right neighbors (Figure 7B).

In Figure 9D, the well-known Pro-right interaction is shown in the very close approach of the Pro Cδ with atom N of the central residue, when this residue is in an A conformation. Figures 9E and 9F show effects of aromatic residues. A left aromatic neighbor (Figure 9E) may have a favorable hydrophobic interaction with the side chain of residue *i*+1, when residue *i* is in a B conformation. A right neighbor of residue *i* that is aromatic can have apparent unfavorable interactions with the carbonyl oxygen of residue *i*−1, as shown in Figure 9F. The position of this oxygen relative to this side chain is determined by φ,ψ of residue *i*, φ of residue *i*+1, as well as the rotamer of the aromatic residue at *i*+1. In several cases studied, when this side chain is in a χ_1_ rotamer of g^−^, which is the most common rotamer of aromatic amino acids, the π orbitals of the ring are in close proximity (about 4.5 Å) to the carbonyl oxygen, as shown in Figure 9F, thus decreasing the tendency of the A conformation when aromatic residues are at position *i*+1. Also, this residue has a φ of −86°, which is consistent with both A and P conformations.

## Discussion

Ramachandran distributions have been produced using many different input data sets and different statistical methods. We have made several choices primarily for the purpose of protein structure prediction, in particular modeling regions of non-regular secondary structure. First, we have used a large input data set of 3,038 proteins with better than or equal to 1.8 Å resolution. Second, we discarded residues with electron density in the bottom 20^th^ percentile [34] in order to remove conformations in potentially mobile parts of the input structures. Third, we derived data sets of all residues not in helix or sheet and a second data set also excluding 3_10_-helix and π-helix. For comparison, we also studied residues in β turns and in coil (i.e., not in secondary structure or turns). Finally, we used hierarchical Dirichlet process methods to develop smooth and statistically reliable Ramachandran distributions for all 20 amino acid types with each of the 20 amino acids as either left or right neighbor. These so-called neighbor-dependent Ramachandran distributions are shown to be useful in loop structure prediction using the Rosetta program.

Many other such statistical analyses φ,ψ data have appeared previously [11], [12], [14], [18], [27], [29], [42], but few are publicly available for use in structure validation or structure prediction. Our analysis differs in a number of respects from the available distributions. For instance, the Richardson group developed smooth φ,ψ densities using adaptive kernel density estimates [16], [17] from a filtered, high-resolution data set [12]. However, their main purpose was structure validation, and their distributions are not residue dependent; that is, all non-Gly,non-Pro,non-pre-Pro residues are treated in one density estimation. They used a quite narrow kernel in their density estimates with all 18 residue types merged into a single, very large data set. It is likely that their density estimates therefore are able to accurately represent sharp changes in the probability density between allowed and disallowed regions. This is quite important for structural validation, as demonstrated by the widespread use of the MolProbity server [6]. Quite usefully, they do provide a separate pre-Pro distribution, since this distribution exhibits the strongest neighbor-dependence, as well as Gly and Pro distributions.

Amir et al. fit cubic splines to kernel density estimates to produce smooth Ramachandran distributions for each amino acid type individually for protein structure prediction [13]. They used a much smaller data set than used here, a set of 850 proteins previously produced by us for development of the backbone-dependent rotamer library [43], [44], and did not examine neighbor effects. Sosnick et al. calculated neighbor-dependent statistics of backbone conformations; however, these are not full Ramachandran densities but proportions in large regions of the φ,ψ space (α, β, polyproline II, etc.) [26].

The Flory isolated-pair hypothesis [32] states that the pair of dihedral angles in protein backbones, φ,ψ, are independent of the conformation of neighboring residues, and by extension the identity of those residues. This idea has been challenged by statistics from the PDB [26], [28], molecular dynamics simulations [27], exhaustive conformational searches and energy calculations [29], and NMR experiments [45]. The results here confirm these earlier investigations and extend them by deriving full Ramachandran probability densities for all residue-neighbor conformations and for different input data sets (the TCBIG, TCB, T, and C sets).

We also suggest some explanations for some of the effects observed. For left neighbors, these effects in some instances are caused by interactions of the side-chain of residue *i*−1 and backbone NH of residue *i*+1, whose relative positions are determined by φ,ψ, of residue *i*, ψ of residue *i*−1, as well as the χ angles of residue *i*−1. For right neighbors, the effects sometimes stem from a complementary interaction – the side chain of residue *i*+1 and the backbone O = C of residue *i*−1, whose relative positions are determined by φ,ψ, of residue *i*, φ of residue *i*+1, and the side-chain conformation of residue *i*+1. In both cases, these interactions can be electrostatic repulsions, hydrogen bonds, or steric, in some cases by blocking access of hydrogen bond donors or acceptors to the backbone. These are commonly described for some residue types in α-helices or β sheets, or capping positions of regular secondary structures, but they also operate in turn and coil conformations of long protein loops.

The key idea of the statistical approach developed here is that more precise estimates of Ramachandran distributions can be found if we examine these distributions in different contexts. This differentiation by context creates a data sparsity problem, in that some contexts may yield very few data points, but, as we have shown, this problem can be addressed effectively within a hierarchical Bayesian framework. Our biochemical knowledge about relatedness can be used to reap further benefits of differentiation of different classes.

The general idea of hierarchical modeling is widespread in Bayesian statistics [46]. It is most common in parametric Bayesian modeling, where parameters are often shared among multiple parametric distributions (e.g., the probabilities of recovery of ill patients are similar if the patients are in the same hospital, have the same doctor, etc.). As we have seen, however, the same basic concepts apply in nonparametric Bayesian modeling. Thus we are able to share statistical strength among multiple multi-modal distributions in which the number of modes in each distribution is unknown a priori. In particular, the hierarchical Dirichlet process allows us to separate Ramachandran distributions according to neighboring residue types and to exploit similarities in these distributions. Moreover, although we have focused on contexts that are defined by amino acid neighborhoods, the same ideas could be used to estimate Ramachandran distributions that are differentiated according to other contextual variables, such as loop structure class (turn, coil, 3_10_-helix, etc.) or neighbor conformational class (A, B, P, etc).

We believe the neighbor-dependent distributions developed here will provide utility in a number of applications in protein structure prediction and structure determination. The appropriate distributions will be specific to each application. We hope that by making them publicly available, their properties may be further explored in various applications.

## Methods

### Data set

We selected a list of 3038 proteins from structures in the Protein Data Bank (PDB) that also had electron densities available from the Uppsala Electron Density Server (EDS) [35]. Using the list of PDB entries with available electron density maps, we entered this list into the PISCES server (http://dunbrack.fccc.edu/pisces) [47], [48] to obtain a subset with resolution better than or equal to 1.7 Å, R-factor ≤0.25, and mutual sequence identity less than 50%. Secondary structure was determined with the program Stride [36], which assigns H (helix), E (sheet), B (bridge), T (Turn), G (3_10_ helix), I (π-helix), and C (coil) to all residues. In order to explore the distributions in longer loop regions, we excluded loops (non-E,H segments) of less than 6 amino acids as well as the first two non-E,H residues following each E or H and the two non-E,H residues preceding E or H. This was done to avoid secondary-structure-capping residues, which have specific distributions in order to break the secondary structure (e.g., N and C cap residues in helices usually have conformations outside the α region; otherwise they would likely be part of the helix).

We used a quality measure of each residue's backbone by calculating the geometric mean of the electron density at backbone atom coordinates as described in previous work [34]. We excluded those residues in the bottom 20^th^ percentile for each residue type from the data. This filter works similarly to a B-factor filter. We use the former because some X-ray structures do not have consistent values for B-factors versus electron density [34].

We created several sets of data for analysis with the HDP procedure:

1. TCBIG set: All residues in non-E,H regions except for the first two and last two of such segments as described above;
2. TCB set: All residues in TCBIG minus residues in 3_10_ and π helices;
3. T set: just those residues in turns;
4. C set: just those residues in coil.

### Hierarchical Dirichlet process

Our approach to modeling neighbor-dependent Ramachandran densities is based on a Bayesian nonparametric density model known as the *hierarchical Dirichlet process (HDP) mixture model* [33]. The HDP approach allows us to subdivide our data into groups defined by a specific central amino acid and by a particular neighboring amino acid. Given these groups, the HDP mixture model produces density estimates for each group in a manner that takes advantage of the commonalities among the groups while allowing each group to exhibit idiosyncratic features.

Before providing a detailed description of the model, let us provide a non-technical overview. Our approach is based on modeling densities such as the Ramachandran distribution as mixtures (i.e., weighted sums) of simple Gaussian component densities. Each such component density is a unimodal bump in the two-dimensional Ramachandran plot, and the overall density is a weighted sum of such bumps. There is a global library of component densities for a single amino acid type, and each particular density (i.e., the Ramachandran density corresponding to a specific right or left neighbor) draws a number of component densities from the global library. The specific details of this model – the locations and orientations of the Gaussian bumps, the number of bumps used in each Ramachandran density, and the pattern of sharing of the bumps between the multiple Ramachandran densities – arise from a Bayesian inference procedure that combines our prior assumptions (the HDP model described below) with the observed data of amino acid dihedral angles in the Ramachandran maps. In essence, the inference procedure based on a Markov chain Monte Carlo procedure finds configurations of the model that are compatible with both the prior and the data.

We now turn to a more technical description of the model. The HDP model can be viewed as a generalization of a simpler model, the Bayesian finite mixture model. Accordingly, we begin with a description of Bayesian finite mixtures and then develop the generalization to the HDP. A classical finite mixture considers a set of *component densities*:
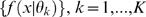
where *x* in this case is the two-dimensional vector of Ramachandran angles, and where 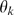 is a parameter vector associated with the *k*th density. For example, *f* might be a Gaussian density, with parameter vector *θ*, representing the mean and covariance matrix. We assume that the number *K* is known and fixed; this assumption will be removed in the HDP approach. We assume that a data point *x_i_* is generated according to the following process:

1. Select an index *k* according to probabilities 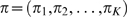, such that 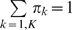

2. Generate a data point *x_i_* by sampling from the distribution 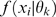.

This process is repeated *N* times, yielding a data set 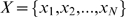. The probability of the 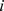
th data point can be written as follows:
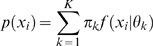
and the overall probability of 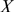 is obtained by taking a product over these probabilities.

In the Bayesian approach to finite mixture models, the parameters π and 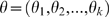 are assumed to be generated from a prior probability distribution. Specifically, in the case of Gaussian mixture models, where 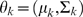 consists of a mean and covariance matrix, a common choice for the prior is
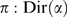


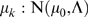


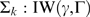
where *Dir* is the *Dirichlet distribution*, *IW* is the *inverse Wishart distribution*, and where *α*, *μ*_0_, Λ, *γ*, and Γ are hyperparameters that are often fixed a priori but also can be inferred from the data.

The next step in our development of the HDP mixture model is the *Dirichlet process (DP) mixture model*, which is a generalization of Bayesian finite mixture models where the number *K* is treated as unknown and to be inferred from the data [20]. This generalization is often described metaphorically in terms of a simple stochastic process known as the *Chinese restaurant process* (CRP). Consider a Chinese restaurant with an infinite number of tables and consider the following seating process. The first customer to arrive in the restaurant sits at the first table with probability one. The second customer then joins that first customer with probability 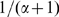 and starts a new table with probability 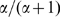, where 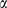 is a parameter. The general rule is that the *n*th customer sits at a table with probability proportional to the number of people already sitting at that table. She may sit at a new table with probability 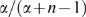.

The CRP determines a clustering or partitioning of the customers. We can turn this clustering process into a mixture model by associating the *k*th table with the *k*th component in a mixture model. In particular, let us assume that the first customer to sit at the *k*th table in the restaurant selects a dish, given by parameter 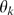, for that table from some prior distribution on the parameters. Each subsequent customer who sits at that table inherits that parameter vector. By viewing the *i*th customer as a data point 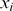 and drawing 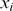 from the distribution 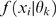, where 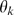 is the parameter vector at the table where customer *i* sits, we obtain a mixture model for generating data.

Note that the number of tables in the CRP, which corresponds to *K* in the finite mixture model, is a random variable that grows as *N* (the number of data points) grows. Indeed, the expected value of the number of occupied tables turns out to scale as log *N* [21]. The DP mixture model is “nonparametric” — the number of parameters grow as we obtain more data.

The inferential problem associated with the DP mixture model is as follows. Given a data set *X*, compute a posterior probability distribution on the allocation of data points to tables and on the parameters associated with the tables. This problem can be solved with a variety of standard methods for posterior inference, including Markov chain Monte Carlo [49], sequential Monte Carlo [50] and variational inference [51]. Although we do not provide details here, it is worth noting that the posterior inference algorithms for DP mixtures are relatively simple; for example, in the Markov chain Monte Carlo methods, we repeatedly revisit each data point and assess which table it should be moved to given the current configuration of all of the other data points. The probability of assigning a point to a table is proportional to the product of the number of data points already at that table and the likelihood of that data point given the parameter vector (or dish) of the table.

We now turn to the *hierarchical Dirichlet process* (HDP) [33]. The HDP can also be described with a restaurant metaphor, in this case by the *Chinese restaurant franchise* (CRF). The problem now is to model the densities associated with each of *M* groups of data; in particular, for a given amino acid, the *M* groups correspond to the set of right or left neighbors. Accordingly, in the CRF there are *M* = 20 restaurants. Data points are categorized as to which group they belong to. A data point (customer) enters the restaurant associated with its group and sits at a table with probability proportional to the number of customers currently sitting at the table. Moreover, the first customer to sit at a table selects a parameter vector for that table. In the CRF metaphor, the parameters are viewed as “dishes,” and the dishes are obtained from a menu that is shared among all of the restaurants. When a dish is selected from the menu a check mark is placed next to that dish. When a new customer goes to the menu to select a dish for her table, she selects a dish with probability proportional to the number of check marks next to that dish and the likelihood of her data point given the parameter vector associated with the dish. Additionally, there is a probability of selecting a new dish.

The global menu implements a sharing of mixture components among the restaurants. Let us consider concretely how this creates a link among multiple density estimation problems. Consider in particular the case of Gaussian mixtures, in which the parameter 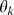 consists of a mean and covariance matrix. When a specific 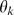 is selected by a customer 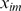 in the *m*th restaurant, this corresponds to a Gaussian bump in the density associated with the *m*th group. Because this parameter vector appears on the global menu, it can then be selected by a customer in one of the other restaurants. This means that that Gaussian bump can also appear in one of the other density models. This allows us to capture commonalities among the groups. Of course, some dishes will only be selected in a single restaurant, and this allows the corresponding group of data to exhibit idiosyncratic features. Tables and dishes are selected in part based on the likelihood of the customer data points given the parameter vectors of the dishes served at each table.

We now describe in greater detail the specifics of the model and estimation procedures used for Ramachandran data. We use the Gaussian mixture HDP to give density estimates of the φ,ψ, dihedral angles conditional on the either the central and left residues (C,L) or the central and right residues (C,R). Since the central residue clearly affects the φ,ψ, angle densities far more than the neighbors, our hierarchy shares features when the central residue is the same and allows for idiosyncratic features for the different neighboring residues. The choice of conditioning on only two residues was motivated by the fact that conditioning on all three residues resulted in very small data sizes that failed to contain sufficient information to capture appropriate features. The HDP estimates were fit independently for each central residue to avoid computational issues. When proline was the central residue, the trans- and cis- configurations were treated as unique, giving a total of 21 possibilities for the central residue and 20 possible neighboring residues.

A natural choice for the component distributions for the φ,ψ, angles would be the bivariate von Mises distribution, an exponential family distribution for angles [52]. Indeed, this distribution was used in earlier work using the Dirichlet process by Lennox et al. [15] However, posterior inference in the bivariate von Mises model is intractable, requiring the computation of an infinite sum of incomplete Bessel functions. Lennox et al. made use of a Gaussian approximation to the von Mises model within the framework of a Metropolis-Hastings algorithm, but this is still complex. Moreover, the major gains from the von Mises model occur near the boundaries of the Ramachandran plot, where the “wrap around” obtained from the von Mises distribution is helpful. We have pursued a simpler approach, where we rotate the data to lie in the rectangle (50°±180°, 90°±180°). This rotation ensured that the density near the boundary is low, and during model fitting, little mass is lost using component densities – in particular Gaussian densitites – which do not wrap around at the boundary. As a post-processing step, the Gaussian mixture components are treated as wrapped Gaussians to ensure that the final density estimate is smooth even at the low density regions at the boundary. This technique yields a fast algorithm that can be used on large data sets.

A conjugate prior was used for the mixture component parameters; specifically, the prior distribution on *μ_k_*, *Σ*_k_ is Normal-Inverse-Wishart with the following parameters:

Mean: μ_0_ = (50°,90°)

Diagonal covariance matrix: Λ = 25^2^ *I*, where *I* is the unit diagonal matrix

Number of pseudo-observations for the mean: κ_0_ = 0.01

Number of pseudo-observations for the variance: ν_0_ = 2.1

The numbers of pseudo-observations for the mean and variance were chosen to be small to give vague priors but large enough so that the prior is proper. An additional prior is placed on the two Dirichlet process α hyperparameters controlling the probability that a new “table” or “dish” in the CRF is sampled. The prior for each was exponential with mean 10.

Density estimates using this HDP model were obtained using Markov Chain Monte Carlo. We used the augmented Gibbs sampler described previously [33]. The mixture component parameters were integrated over, and only the sufficient statistics, the sample mean and covariance, were retained. After a burnin of 10,000 samples, an additional 50,000 samples were drawn and then thinned, with every 50^th^ sample retained. For each sample the φ,ψ, density was evaluated on a grid of 288×288 values. These 1000 density samples were then averaged to obtain the final density estimates.

### Approximating the distributions with cubic splines

Due to the large size of these grids, we fit a cubic tensor spline with 72×72 knots to reduce the size of the representation while providing an excellent approximation to the original fit. A degree *d* spline approximation to a function uses a piecewise polynomial of degree *d* to approximate the function. The location of each piece of the piecewise polynomial is determined by the knots, and the piecewise polynomial is constrained to have *d*−1 continuous derivatives. Thus, the compactness of the approximation is controlled by the number of knots, and the smoothness is controlled by the degree *d*. This spline representation has the additional advantage of being very fast to compute since evaluating a polynomial requires only a few basic multiplication and addition operations. Since the angle data are periodic, we also enforced the smoothness constraint at the boundaries at ±180°.

A two dimensional tensor spline is one where each “piece” of the piecewise polynomial is of the form 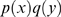 where 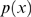 and 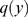 polynomials. Once the knots are defined, a 72×72 uniform grid in our case, a regression spline is easily fit by minimizing the squared error to a target, a 288×288 grid of log density estimates in our case. Compared to linear interpolation on a 72×72 grid representation, which consumes an equivalent amount of memory, the spline approximation improved the approximation error, as measured by Kullback-Leibler divergence, from 0.22 to 0.004.

### Hellinger distance

We used the Hellinger distance to determine the similarities of different Ramachandran distributions. For two probability distributions, 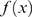 and 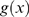, the Hellinger distance, *H* is calculated from the following equation:
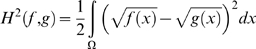
where the integral is taken over the domains of *f* and *g*. *H* satisfies the expression 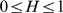.

### Rosetta loop predictions

We used a loop-prediction data set described by Soto et al. [38], consisting of 290 loops (the original set consisted of 293 targets but Rosetta was unable to complete three of them. This set consisted of loops from several previous benchmarks, with a total of 62, 56, 40, 54, 39, and 39 loops of lengths 8, 9, 10, 11, 12, and 13 residues respectively.

Loop modeling was performed with Rosetta2.3.0, modified to use the neighbor-dependent Ramachandran distributions. We used the standard pose-based loop modeling protocol built into Rosetta [53], using a fixed backbone and side chains for all residues except those in the loop region to be predicted. We generated 2000 decoys for loops of length 8 and 9, 5000 decoys for loop lengths 10–12, and 8000 decoys for loop length of 13. For each individual loop, a random starting conformation is constructed by arbitrarily inserting fragments in the loop region. The fragment library was generated using the standard Rosetta fragment generating tools, i.e. searching with the query sequence of each loop against representative PDB structures skipping homologous structures (-nohoms option).

Once the initial conformation was built, the simulation was performed in two steps. In the first (low-resolution) step, the side chains were represented by centroid atoms. A series of Monte Carlo perturbation steps followed by loop closure using cyclic coordinate descent (CCD) [54] and line energy minimization were performed. The conformation perturbation was done by inserting three-residue and one-residue fragments into the loop region. In the second (high-resolution) step, all atoms including hydrogen atoms were explicitly represented. The perturbation was done by introducing small random changes to one or more backbone torsions angles, followed by CCD closure, and Davidson-Fletcher-Powell minimization. Repacking of all the loop side chains was performed after every 20 cycles as well as at the end of the overall simulation.

The full command line for loop modeling was:

rosetta.gcc 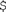
serial 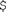
entry 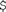
chain -pose -loops -fa_input -fold_with_dunbrack -fast -fix_natsc -ramaneighbors 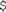
type -rama_file 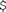
ramafile -pose_silent_out -pose_loops_file 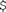
entry
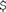
chain.loop -s 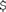
entry
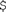
chain.pdb -nstruct 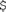
nStructs

where variables with “$” signs were defined within a loop:

- $serial = any two-letter string
- $entry = four-letter PDB name
- $chain = 1-letter chain identifier
-ramaneighbors $type = “none” uses neighbor-independent distributions
-ramaneighbors $type = “both” uses neighbor-dependent distributions
-rama_file $ramafile = name of Ramachandran distribution file used
-nstruct $nStructs = number of decoys that will be generated.
-pose_silent_out: Use compressed output for decoys.
-fa_input: Input fullatom coordinates (implies -fa_ouput.)
-pose: The pose version of the loop modeling protocol using -fold_with_dunbrack. It supports multiple loops and will fold them in centroid in the order of input. After each move step, full-atom refinement is performed It works with -trim, -fix_natsc options. And use -fast to speed up the protocol by reducing the number of cycles of trial.
-fold_with_dunbrack: An alternative loop modeling protocol which combines fragment insertion and cyclic coordinate descent loop closure protocol during each step
-loops: Manipulate and try to form loop secondary structures.
-fast: Use fast protocols
-fix_natsc: Use native rotamers for template, i.e., the non-loop regions
-pose_loops_file <file>: Specify a single file to use as the loop file

The command-line options -ramaneighbors and -rama_file were added to this version of Rosetta, specifically for using the neighbor-dependent Ramachandran distributions.

### Ramachandran distributions dependent on both left and right neighbors

To obtain density estimates of φ,ψ, for the central residue conditional on all three residue types, the following model can be used to combine the HDP density estimates which are conditional only on central/left or central/right residue pairs.
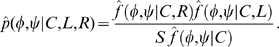
where *S* is a normalizing constant obtained by integrating the expression on the right hand side (without the *S*). This estimate is the plug-in estimator for the full conditional probability given the assumption that the identity of the left and right residues are independent given φ,ψ,. For most residues, *S* was near 1 but for some residues and some neighbors, in particular proline, *S* was as low as 0.5 and as high as 1.5. The normalization is therefore important.

### Regions of the Ramachandran map

In order to characterize the effects of neighbors on populations on different regions of the Ramachandran map, we divided the φ,ψ space into non-overlapping bins as follows, for the α, β, polyproline II, left-handed, and γ conformations, respectively:

A: −200°≤φ<0°, −120°<ψ≤40°, 90°≤ω≤270°

B: −90°≤φ<0°, 40°<ψ≤240°, 90°≤ω<270°

P: −200°≤φ<−90°, 40°<ψ≤240°, 90°≤ω<270°

L: 0°≤φ<160°, −90°<ψ≤110°, 90°≤ω<270°

E: 0°≤φ<160°, 110°<ψ≤270°, 90°≤ω<270°

cis: −90°≤ω<90°

### Figures

Density plots were produced in Matlab (the Mathworks, Inc., Natick, MA, USA). The multi-dimensional scaling and QQ plots were performed in R (the R Foundation for Statistical Computing, Vienna, Austria). Protein images were produced in PyMol (DeLano Scientific, Palo Alto, CA, USA).

### Availability

All distributions are freely available to non-profit research groups at this address: http://dunbrack.fccc.edu/hdp.
